# Repurposing Vandetanib plus Everolimus for the Treatment of *ACVR1*-Mutant Diffuse Intrinsic Pontine Glioma

**DOI:** 10.1158/2159-8290.CD-20-1201

**Published:** 2021-09-22

**Authors:** Diana M. Carvalho, Peter J. Richardson, Nagore Olaciregui, Reda Stankunaite, Cinzia Lavarino, Valeria Molinari, Elizabeth A. Corley, Daniel P. Smith, Ruth Ruddle, Adam Donovan, Akos Pal, Florence I. Raynaud, Sara Temelso, Alan Mackay, John P. Overington, Anne Phelan, David Sheppard, Andrew Mackinnon, Bassel Zebian, Safa Al-Sarraj, Ashirwad Merve, Jeremy Pryce, Jacques Grill, Michael Hubank, Ofelia Cruz, Andres Morales La Madrid, Sabine Mueller, Angel M. Carcaboso, Fernando Carceller, Chris Jones

**Affiliations:** 1 Division of Molecular Pathology, Institute of Cancer Research, London, United Kingdom.; 2 BenevolentAI, London, United Kingdom.; 3 Laboratory of Molecular Oncology, Hospital Sant Joan de Déu, Barcelona, Spain.; 4 Molecular Diagnostics, Royal Marsden Hospital NHS Trust, Sutton, United Kingdom.; 5 Children & Young People's Unit, Royal Marsden Hospital NHS Trust, Sutton, United Kingdom.; 6 Division of Cancer Therapeutics, Institute of Cancer Research, London, United Kingdom.; 7 Medicines Discovery Catapult, Alderley Edge, United Kingdom.; 8 Atkinson Morley Regional Neuroscience Centre, St George's Hospital NHS Trust, London, United Kingdom.; 9 Department of Neurosurgery, Kings College Hospital NHS Trust, London, United Kingdom.; 10 Department of Clinical Neuropathology, Kings College Hospital NHS Trust, London, United Kingdom.; 11 Institute of Neurology, University College London Hospitals, London, United Kingdom.; 12 South West London Pathology, St George's Hospital NHS Trust, London, United Kingdom.; 13 Department of Pediatric and Adolescent Oncology and INSERM Unit U891, Team “Genomics and Oncogenesis of Pediatric Brain Tumors,” Gustave Roussy and University Paris-Saclay, Villejuif, France.; 14 Paediatric Oncology, Neuro-Oncology Unit, Hospital Sant Joan de Déu, Barcelona, Spain.; 15 University Children's Hospital, Zurich, Switzerland.; 16 University of California, San Francisco, San Francisco, California.; 17 Division of Clinical Studies, The Institute of Cancer Research, London, United Kingdom.

## Abstract

Somatic mutations in *ACVR1* are found in a quarter of children with diffuse intrinsic pontine glioma (DIPG), but there are no ACVR1 inhibitors licensed for the disease. Using an artificial intelligence–based platform to search for approved compounds for *ACVR1*-mutant DIPG, the combination of vandetanib and everolimus was identified as a possible therapeutic approach. Vandetanib, an inhibitor of VEGFR/RET/EGFR, was found to target ACVR1 (*K*_d_ = 150 nmol/L) and reduce DIPG cell viability *in vitro* but has limited ability to cross the blood–brain barrier. In addition to mTOR, everolimus inhibited ABCG2 (BCRP) and ABCB1 (P-gp) transporters and was synergistic in DIPG cells when combined with vandetanib *in vitro*. This combination was well tolerated *in vivo* and significantly extended survival and reduced tumor burden in an orthotopic *ACVR1*-mutant patient-derived DIPG xenograft model. Four patients with *ACVR1*-mutant DIPG were treated with vandetanib plus an mTOR inhibitor, informing the dosing and toxicity profile of this combination for future clinical studies.

**Significance:**

Twenty-five percent of patients with the incurable brainstem tumor DIPG harbor somatic activating mutations in *ACVR1*, but there are no approved drugs targeting the receptor. Using artificial intelligence, we identify and validate, both experimentally and clinically, the novel combination of vandetanib and everolimus in these children based on both signaling and pharmacokinetic synergies.

*This article is highlighted in the In This Issue feature, p. 275*

## Teaser

Combined vandetanib and everolimus was identified by artificial intelligence as a potential therapy for *ACVR1*-mutant DIPG, as they synergize in vitro and extend survival *in vivo*, with preliminary testing in four children suggesting this combination as a feasible clinical option.

## INTRODUCTION

Diffuse intrinsic pontine glioma (DIPG) is an incurable infiltrating glioma of the brainstem in children, with a median overall survival (OS) of 9 to 12 months (1–3). Radiotherapy is the only treatment paradigm that provides a therapeutic response, although more than 90% of children relapse and die of their disease within 2 years (1, 3, 4). This tumor type comprises approximately 10% of all pediatric brain tumors, and outcomes remain poor, partly due to its unique biology, as evidenced by the high prevalence (>80%) of lysine-to-methionine substitutions at position 27 (K27M) in the *H3F3A*, *HIST1H3B*, and *HIST1H3C* genes (5, 6).

Mutations commonly associated with the H3.1K27M substitutions include those in the *ACVR1* gene, which is mutated in approximately 25% of all DIPG tumors. These mutations have been reported at a younger age of diagnosis and with a slightly prolonged OS in children with DIPG (7–10). At least nine such mutations have been recognized predominantly within or close to the tyrosine kinase domain of the receptor (11). These mutations result in an abnormal sensitivity to activin A (12) and/or constitutive receptor activation even in the absence of activating ligands (13). We (12) and others (14) have recently demonstrated that ACVR1 inhibition by selective inhibitors inhibits the growth of orthotopic tumors bearing *ACVR1* mutations. However, the clinical development of these and related compounds still remains to be undertaken before clinical trials can take place.

One way of accelerating the discovery of treatments of DIPG is the repurposing of currently approved drugs, singly or in combination. This concept has to date focused on unbiased screens of such approved compound libraries and by doing so has identified important novel therapeutic options for clinical use, particularly the histone deacetylase inhibitor panobinostat used alone (15) or combined with the proteasome inhibitor marizomib (16). Other candidates, such as the IGF1R [and other receptor tyrosine kinase (RTK)] inhibitor BMS-754807 (17) and the FGFR inhibitor ponatinib (18), along with disulfiram and MDM2 and Bcl2 inhibitors (19), are also being explored. An alternative approach that enables the exploitation of the enormous amount of scientific data available to researchers is to use artificial intelligence (AI) methods, which are only recently being harnessed for cancer drug discovery (20).

To facilitate such an AI-augmented approach for *ACVR1*-mutant DIPG, BenevolentAI used an internally developed biomedical knowledge graph. This graph contains in excess of a billion provenanced relationship edges linking disease, biological mechanisms, and drug targets that are derived automatically from both structured and unstructured data. As an example of the power of such an approach, this platform was queried in early 2020 in the search for an approved drug that could be used in the treatment of the COVID-19 pandemic. In early 2020, this resulted in the identification of the anti-inflammatory drug baricitinib as a potential therapeutic capable of reducing viral infectivity and the associated overexuberant inflammation seen in this disease (21–24). As a result, baricitinib has been used in multiple investigator-led clinical trials and in two major randomized clinical trials, one recently published (25).

For *ACVR1*-mutant DIPG, we used this knowledge graph to identify compounds that could be used to inhibit ACVR1, with a particular focus on the likely ability to identify compounds that would cross the blood–brain barrier. In the present study, we demonstrate that two approved drugs used in combination, vandetanib and everolimus, worked synergistically *in vitro* and *in vivo* to inhibit the growth of tumors bearing *ACVR1* mutations, and we report on the early clinical experience in children with *ACVR1*-mutant DIPG. Although careful management of side effects and prior treatment will be essential, such an approach may form the basis for future formal clinical trials of the combination in patients with this genotype.

## RESULTS

### AI-based Identification of Approved Therapeutics

The BenevolentAI knowledge graph contains more than 40 million documents and more than 1 billion relationship edges. It contains diseases, biological tissues, mechanisms and pathways, gene ontology processes, genes, proteins, as well as drugs, biologics, and small molecules (26). To find approved drugs with activity against ACVR1 that could be rapidly explored in the clinical context, we searched the knowledge graph for compounds that may have inhibitory effects on the ALK2 protein. Inherent within this approach was the necessity to find drugs with known central nervous system (CNS) penetration. In total, 856 compounds that inhibit ALK2 enzyme activity were identified and then prioritized according to potency (IC_50_) and whether they had been approved by the regulators for medicinal use. No approved drugs were identified that would be predicted to penetrate the CNS at sufficient concentrations to inhibit ALK2 using therapeutic doses. The four most promising compounds in terms of nanomolar ALK2 efficacy (vandetanib, dasatinib, crizotinib, and nintedanib) had insufficient brain exposures as a result of being substrates of the blood–brain barrier efflux transporters ABCB1 (P-gp) and/or ABCG2 (BCRP; refs. 27, 28).

**Figure 1.**
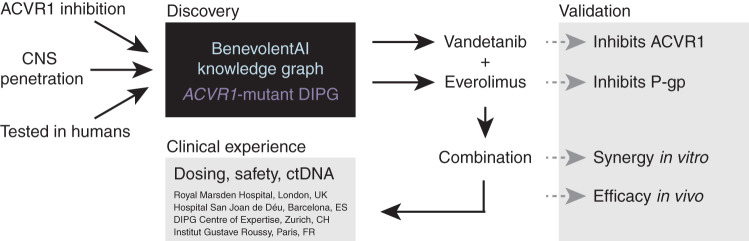
Schema for AI-based identification of a repurposed drug combination strategy for *ACVR1*-mutant DIPG. The BenevolentAI knowledge graph was employed to identify approved drugs with potential potency against ACVR1 and sufficient CNS penetration to be a rational therapy for children with DIPG. Vandetanib and everolimus were validated to inhibit ACVR1 and P-gp, respectively, and the combination was found to be synergistic *in vitro* and prolong survival *in vivo*, leading to the clinical use of this combination in four expert pediatric neuro-oncology centers in Europe. ctDNA, circulating tumor DNA.

We therefore queried whether a combination of a putative ACVR1 inhibitor and another drug known to interfere with the drug transporter–mediated efflux could be found that had been previously shown to be safe in humans. In addition, compounds were assessed for possible feedback mechanisms that themselves could be blunted by the combination of inhibitors. The AI system output suggested combinations of vandetanib or dasatinib with mTOR/FKBP12 inhibitors such as everolimus or sirolimus. Because everolimus has the higher affinity for mTOR/FKBP12 and has already been approved for cancer treatment, we selected it as the candidate mTOR/ABC transporter inhibitor. Both dasatinib and vandetanib are being used in clinical trial combinations with everolimus, suggesting both combinations are already being considered in children and adults (NCT03352427 and NCT01582191). The sequence of the logic is shown in Fig. 1.

### Validation of Selected Agents

**Figure 2.**
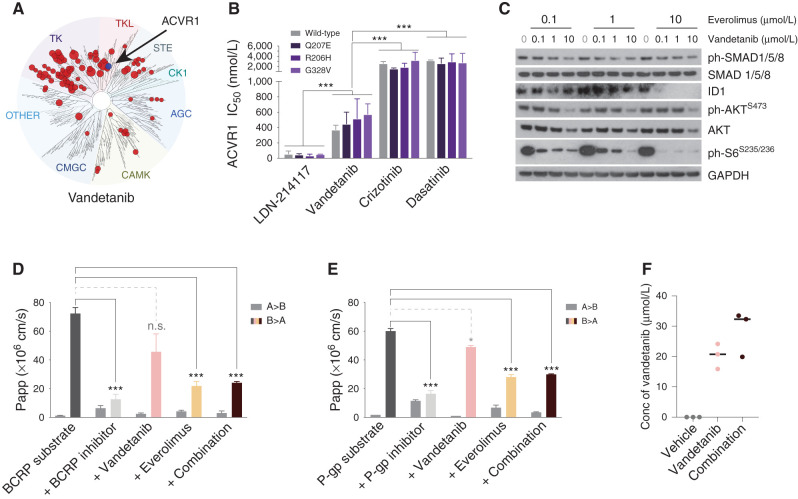
Validation of vandetanib as an ACVR1 inhibitor and everolimus as a P-gp inhibitor. **A,** Kinome selectivity map for vandetanib at 10 μmol/L, with red circles reflecting kinases inhibited by >65%. ACVR1 is labeled. The diameter of the circles is inversely proportional to the percentage of kinase activity remaining in the presence of an inhibitor. Taken from the Harvard Medical LINCS KINOMEscan database (https://lincs.hms.harvard.edu/). **B,** Bar plots showing the IC_50_ for various compounds against wild-type (gray) and mutant ACVR1: Q207E, R206H, and G328V (shades of purple). **C,** Western blot of *ACVR1*^R206H^ HSJD-DIPG-007 cells treated with increasing concentrations of vandetanib in the presence of 0.1 μmol/L, 1.0 μmol/L, or 10 μmol/L everolimus. GAPDH is the loading control. **D,** Bar plots showing apparent permeability (Papp) of a BCRP substrate across a bidirectional cell monolayer (A–B and B–A) in the presence of various inhibitors. **E,** Bar plots showing apparent permeability (Papp) of a P-gp substrate across a bidirectional cell monolayer (A–B and B–A) in the presence of various inhibitors. Vandetanib, pink; everolimus, light orange; combination, dark red. Error bars represent SD of the mean. ***, *P* < 0.0001, ANOVA. n.s., not significant. **F,** Dot plot of vandetanib concentration (Conc) in the brains of normal mice treated with vehicle control (gray), vandetanib (pink), or the combination (dark red), as assessed by mass spectrometry. The black horizontal line represents the median.

Dasatinib as a multikinase inhibitor had been previously suggested as an ACVR1 inhibitor (29), as had the ALK/MET inhibitor crizotinib (30). To the best of our knowledge, vandetanib, used clinically as a VEGFR/EGFR/RET inhibitor, had not. Publicly available KINOMEscan data (31) for vandetanib indicated a *K*_d_ for ACVR1 of 150 nmol/L (in comparison to EGFR of 4.8 nmol/L, RET of 14.0 nmol/L, and VEGFR1 of 260 nmol/L; Fig. 2A). We next used biochemical assays to assess the ability of these compounds to inhibit the ALK2 protein, along with the most common mutations found in DIPG, in comparison to the tool ACVR1 inhibitor LDN-214117. There were no significant differences between IC_50_ values against wild-type or mutant ALK2 (including the constitutively activating mutation Q207E) for any of the agents tested (Fig. 2B). Vandetanib, however, was fivefold to sixfold more potent against ACVR1 than either dasatinib or crizotinib (IC_50_ 466 nmol/L vs. 2,894 nmol/L and 2,334 nmol/L, respectively, *P* < 0.0001, ANOVA), albeit also an average of approximately ninefold less potent than LDN-214117 (IC_50_ 466 nmol/L vs. 51 nmol/L, *P* < 0.0001, ANOVA; Supplementary Fig. S1).

There was a small but significant enhanced difference in cell viability *in vitro* in *ACVR1*-mutant compared with wild-type patient-derived DIPG cells treated with vandetanib (approximately twofold, *P* = 0.0272, *t* test; Supplementary Fig. S2A), which was accompanied by a concentration-dependent inhibition of phosphorylated SMAD1/5/8 in addition to the expected effect on phosphorylated AKT (Ser473) at the highest dose in ACVR1^R206H^ HSJD-DIPG-007 cells (Supplementary Fig. S2B). These effects were not observed with everolimus (Supplementary Fig. S2C), although there was a reduction in ID1 (Supplementary Fig. S2D). Notably, when the two drugs were combined, expression of both ID1 and the mTOR target phospho-S6 kinase was ablated when treated with even the lowest doses of vandetanib in the presence of 10 μmol/L everolimus (Fig. 2C).

Next, everolimus was explored for its ability to inhibit key transporter pumps in an *in vitro* Caco-2 permeability assay. Although vandetanib showed weak interactions with P-gp and BCRP, everolimus significantly decreased the B–A apparent permeabilities of control substrates with both transporters (*P* < 0.0001, *t* test for both BCRP and P-gp; Fig. 2D and E). The addition of vandetanib made no effect to either when tested in combination. In non–tumor-bearing mice *in vivo*, treatment with 10 μmol/L everolimus increased the median brain concentration of vandetanib by 56% compared with vandetanib alone, although this failed to reach statistical significance due to one mouse failing to show an effect (*P* = 0.0854, *t* test; Fig. 2F).

### Efficacy of Combined Vandetanib and Everolimus in *ACVR1*-Mutant DIPG Models

**Figure 3.**
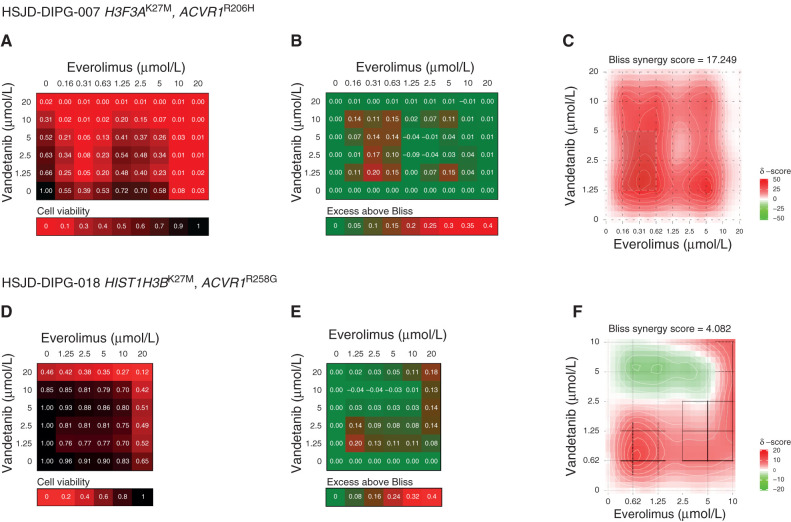
Synergy of combined vandetanib and everolimus *in vitro*. **A,** Cell viability matrix for *ACVR1*^R206H^ HSJD-DIPG-007 cells treated with distinct combinations of vandetanib (*y*-axis) and everolimus (*x*-axis) ranging from 0 to 20 μmol/L. **B,** Excess above Bliss matrix for *ACVR1*^R206H^ HSJD-DIPG-007 cells treated with distinct combinations of vandetanib (*y*-axis) and everolimus (*x*-axis) ranging from 0 to 20 μmol/L. **C,** Bliss synergy map for *ACVR1*^R206H^ HSJD-DIPG-007 cells treated with distinct combinations of vandetanib (*y*-axis) and everolimus (*x*-axis) ranging from 0 to 10 μmol/L. Heat maps colored according to the keys provided. **D,** Cell viability matrix for *ACVR1*^R258G^ HSJD-DIPG-018 cells treated with distinct combinations of vandetanib (*y*-axis) and everolimus (*x*-axis) ranging from 0 to 20 μmol/L. **E,** Excess above Bliss matrix for *ACVR1*^R258G^ HSJD-DIPG-018 cells treated with distinct combinations of vandetanib (*y*-axis) and everolimus (*x*-axis) ranging from 0 to 20 μmol/L. **F,** Bliss synergy map for *ACVR1*^R258G^ HSJD-DIPG-018 cells treated with distinct combinations of vandetanib (*y*-axis) and everolimus (*x*-axis) ranging from 0 to 10 μmol/L. Heat maps colored according to the keys provided.

Having validated the key tenets of the individual drugs, we next sought to explore the effects of combining them on patient-derived *ACVR1*-mutant DIPG cells. We used the Bliss independence model to assess the combination of vandetanib and everolimus in terms of cell viability in *H3F3A*^K27M^, *ACVR1*^R206H^–mutant HSJD-DIPG-007 (Fig. 3A–C) and *HIST1H3B*^K27M^, *ACVR1*^R258G^–mutant HSJD-DIPG-018 (Fig. 3D–F). In both models, profound effects were seen, especially at low doses of both drugs (Fig. 3A and D). This was more formally evaluated by calculating the excess above Bliss scores for each pairwise combination, identifying likely synergistic areas within the combinatorial matrix (Fig. 3B and E). A summary synergy score was calculated as the average excess response due to drug interactions above expectation, with the resulting value of 17.25 indicating a high degree of formal synergy in HSJD-DIPG-007 (Fig. 3C) and a more restricted synergistic interaction at lower doses of vandetanib in HSJD-DIPG-018 (overall score 4.08, peak value 7.72; Fig. 3F).

**Figure 4.**
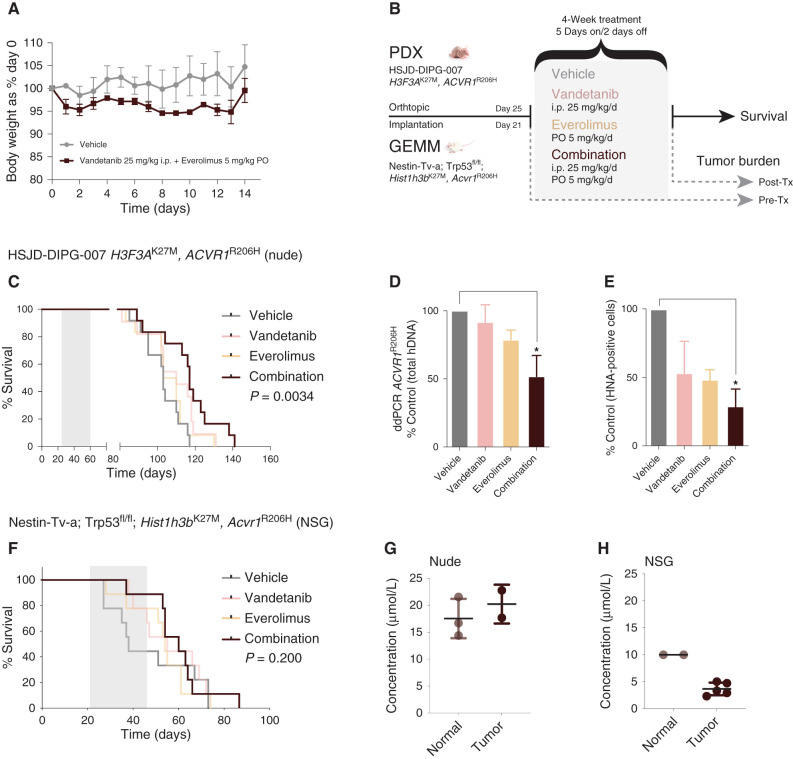
Efficacy of combined vandetanib and everolimus *in vivo*. **A,** Tolerability in NOD.SCID mice exposed to daily oral treatment with the combination of vandetanib and everolimus over 14 days, as assessed by body weight relative to day 0. Mean and SD of three mice per group are plotted. PO, oral administration. **B,** Schema for *in vivo* efficacy (survival) and tumor burden experiments in orthotopic PDX and GEMM allografts of mutant ACVR1-driven DIPG. Tx, treatment. **C,** Survival curves for mice (*n* = 16–22 per group) bearing HSJD-DIPG-007 orthotopic xenografts, treated with vandetanib (pink), everolimus (light orange), or the combination (dark red), compared with vehicle-treated controls (gray). **D,** Bar plot quantifying tumor burden as assessed by ddPCR for *ACVR1*^R206H^ in mice treated with vandetanib (pink), everolimus (light orange), or the combination (dark red), expressed as a percentage of vehicle-treated controls (gray). **E,** Bar plot quantifying cellularity by human nuclear antigen (HNA)–positive cells in mice treated with vandetanib (pink), everolimus (light orange), or the combination (dark red), expressed as a percentage of vehicle control (gray). Mean and SD plotted. *, *P* < 0.05, adjusted *t* test. **F,** Survival curves for mice (*n* = 16–22 per group) bearing Nestin-Tv-a; Trp53^fl/fl^; *Hist1h3b*^K27M^, Acvr1^R206H^ orthotopic allografts, treated with vandetanib (pink), everolimus (light orange), or the combination (dark red), compared with vehicle-treated controls (gray). **G,** Dot plot of vandetanib concentration in the normal brains and engrafted tumors of nude mice treated with combined vandetanib and everolimus, as assessed by mass spectrometry. The black horizontal line represents the median. **H,** Dot plot of vandetanib concentration in the normal brains and engrafted tumors of NSG mice treated with combined vandetanib and everolimus, as assessed by mass spectrometry. The black horizontal line represents the median.

Prior to assessing the efficacy of the combination *in vivo*, we ensured that combined vandetanib and everolimus was well tolerated in non–tumor-bearing mice. There was no reduction in body weight outside allowed parameters, and animals remained healthy during the 14-day course (Fig. 4A). We used two distinct DIPG mouse models for testing the combined treatment—a patient-derived xenograft (PDX) model of HSJD-DIPG-007 (12) and an allograft of cells derived from a Nestin-Tv-a; Trp53^fl/fl^; Hist1h3b^K27M^, Acvr1^R206H^ genetically engineered mouse model (GEMM; ref. 14), both implanted orthotopically. Mice were treated with either agent alone or in combination, with a separate pre–post treatment arm to assess tumor burden (Fig. 4B). After a 4-week schedule of 5 days on, 2 days off treatment, combined vandetanib and everolimus was found to extend survival by 2 weeks in the HSJD-DIPG-007 PDX model compared with control (117 days combination vs. 103 days, 14%, *P* = 0.00072, median test), in contrast with either agent alone (log-rank *P* = 0.0034, corrected for multiple testing; Fig. 4C). Similarly, the combination was found to significantly reduce tumor burden over the course of the 4-week treatment, as assessed by both droplet digital PCR (ddPCR) of the ACVR1^R206H^ mutation (ANOVA *P* = 0.0197; Fig. 4D) and IHC staining of human nuclear antigen (ANOVA *P* = 0.0017, both corrected for multiple testing; Fig. 4E; Supplementary Fig. S3A). In the GEMM allografts (Supplementary Fig. S3B), we observed an extension of median survival of 60 versus 38 days (58%, *P* = 0.0375, median test), but this failed to reach formal statistical significance in survival analysis (log-rank *P* = 0.200), with some unexpectedly longer-term survivors in the vehicle-treated arm (Fig. 4F). Measuring tumor penetration of vandetanib when combined with everolimus in the different murine backgrounds used, we noted significantly higher levels (>15 μmol/L) in both normal brain and PDX in nude mice (Fig. 4G) compared with the normal and GEMM allograft in NSG mice (10 μmol/L and <5 μmol/L, respectively; Fig. 4H), although still in excess of *in vitro* GI50 values.

### Clinical Experience in Patients with *ACVR1*-Mutant DIPG

Four children with DIPG for whom *ACVR1* mutations were confirmed in stereotactic tumor biopsy specimens and/or peripheral blood were treated with vandetanib and an mTOR inhibitor. Three of them have annotated clinical details and treatment-related toxicities (Tables 1 and 2). A fourth case of DIPG received vandetanib and everolimus at relapse and was subsequently lost to follow-up.

**Table 1.** Demographics of three children with newly diagnosed *ACVR1*-mutant DIPG treated with vandetanib and mTOR inhibitors upfront or at relapse

Case	Age, y	Sex	Histology	Mutations	RT, Gy/*n*	Timing of combination	Cycles	Previous therapies	EFS months, Dx/combination	OS months, Dx/combination	Status
1	7	Female	DMG	*HIST2H3C* p.K27M	54/30	Upfront	7	Everolimus	11/6.5	20/9	Deceased
				*ACVR1* p.G328V							
				*BCOR* p.Met1020fs							
2	4	Female	DMG	*HIST1H3B* p.K27M	54/30	Upfront	1	Bevacizumab	5/1	9/5	Deceased
				*ACVR1* p.G328E							
3	4	Female	DMG	*HIST1H3B* p.K27M	54/30	Relapse	1	Re-RT, bevacizumab, sirolimus	8/0.7	16/0.7	Deceased
				*PIK3CA* p.E542K							
				*PIK3CA* p.E542K							

NOTE: Cases 1 and 3 were treated with vandetanib/everolimus; case 2 was treated with vandetanib/sirolimus.

Abbreviations: DMG, diffuse midline glioma; Dx, diagnosis; EFS, event-free survival; re-RT, reirradiation; RT, radiotherapy.

**Table 2.** Summary of toxicities graded as per Common Terminology Criteria for Adverse Events version 4.03 related to vandetanib and/or mTOR inhibitors in three children with newly diagnosed *ACVR1*-mutant DIPG for a total of 9 cycles of vandetanib and 12 cycles of mTOR-inhibitor (11 cycles of everolimus and 1 cycle of sirolimus)

Adverse event	Grade 1	Grade 2	Grade 3	Grade 4	Grade 5	Total events
Hematologic						
Decreased leukocytes	0	1	0	0	0	1
Lymphopenia	2	1	0	0	0	3
Neutropenia	0	4	0	0	0	4
Increased APTT	1	0	0	0	0	1
Gastrointestinal						
Mouth ulcer	1	0	0	0	0	1
Diarrhea	3	0	0	0	0	3
Metabolic/laboratory						
Elevated cholesterol	1	2	0	0	0	3
Increased creatinine	2	0	0	0	0	2
Increased ALT	3	0	0	0	0	3
Hypophosphatemia	1	0	0	0	0	1
Hyperglycemia	1	0	0	0	0	1
Proteinuria	2	0	0	0	0	2
Cardiovascular						
Prolonged QTc interval	2	0	0	0	0	2
Hypertension	1	0	1	0	0	2
Intracranial hemorrhage (intratumoral)	0	1	0	0	1	2

Abbreviations: ALT, alanine aminotransferase; APTT, activated partial thromboplastin time.

#### Case 1

**Figure 5.**
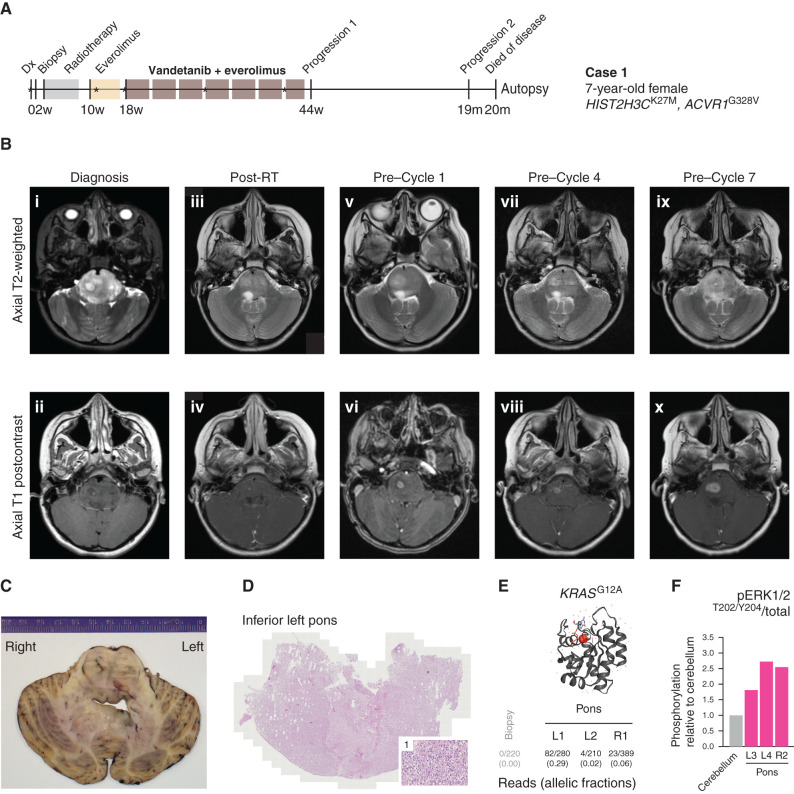
Clinical experience of combined vandetanib and everolimus in a 7-year-old girl with ACVR1-mutant DIPG (case 1). **A,** Timeline summarizing the clinical history of case 1, treated at the Royal Marsden Hospital, London, United Kingdom. *Imaging scans presented in **B**. Dx, diagnosis. **B,** Sequential head MRI scans: diagnostic MRI showing diffuse heterogenous tumor infiltration and anatomic distortion of the pons, including partial invagination of the basilar artery (i) with a single focus of intratumoral enhancement in the right central pons (ii); MRI scan 6 weeks after radiotherapy (RT) showed considerable reduction in tumor infiltrative bulk (iii) with the small focus of enhancement persisting in the right pons (iv); the MRI scan after three cycles of everolimus single agent constituted also the baseline scan prior to starting vandetanib/everolimus, and it showed increased T2 signal abnormality in the right pons (v) associated with mildly increased focal enhancement (vi); the MRI scans after three cycles (vii, viii) and six cycles (ix, x) of vandetanib/everolimus showed modest enlargement and progressive infiltration of the right pons (vii, ix) with concomitant increase of the associated focal enhancement (viii, x) but nevertheless with relatively stable appearances/lack of progression elsewhere. The child started cycle 7 of vandetanib/everolimus but then developed worsening symptoms (slurred speech, ataxia), and treatment was permanently discontinued 11 months after initial diagnosis. No further systemic treatment or reirradiation was administered. The child could be weaned off steroids completely, and her balance and activity levels improved significantly. Eight months after discontinuation of vandetanib/everolimus, she developed further clinical and radiologic progression (local and metastatic) and died a month later (OS, 20 months). **C,** Macroscopic transverse cross section of pons and cerebellum from the brain autopsy specimen from case 1. **D,** Hematoxylin and eosin–stained image from the inferior left pons (×40), with a higher magnification inset (×200) of region 1, showing diffuse infiltration of tumor cells. **E,** Protein structure representation of *KRAS* showing mutant G12A residue (shaded red), generated in COSMIC-3D (https://cancer.sanger.ac.uk/cosmic3d/). Allelic fractions are provided for mutant reads identified in distinct areas of the pons taken at autopsy. **F,** MAPK pathway activation in distinct autopsy regions measured by quantitative capillary electrophoresis for phospho-ERK1/2^T202/Y204^, plotted as a ratio of respective phosphorylated/total protein compared with normal cerebellum.

A 7-year-old female was diagnosed with DIPG in May 2019 under the South Thames Paediatric Neuro-Oncology Team (London, United Kingdom). She was enrolled in the BIOMEDE trial (NCT02233049) and underwent a biopsy but failed screening because of elevated alanine aminotransferase (grade 3). Standard focal photon beam radiotherapy was administered off trial. She then received three cycles of single-agent everolimus on a compassionate use basis. Following identification of an *ACVR1*^G328V^ mutation (Fig. 5A), vandetanib was added on a compassionate use basis in combination with everolimus up to a total of seven cycles. The dosing schedule of this combination was carefully defined based on published evidence of vandetanib ± dasatinib in DIPG (28, 32) and a phase I trial of vandetanib/everolimus in adults with advanced/metastatic lung cancer (33, 34): vandetanib 65 mg/m^2^/d and everolimus 4 mg/m^2^/d (34). The frequency and severity of toxicities were increased compared with single-agent everolimus but with acceptable tolerance to the point that the child was able to resume school part-time. The treatment was halted temporarily during cycles 1 and 5 due to transient clinical deterioration (increased facial droop, slurred speech, and unsteadiness). On both occasions, vandetanib/everolimus was restarted at the same doses, with stable MRI findings and following improvement after a short course of steroids. During cycle 6, she developed grade 3 hypertension unrelated to steroids. Vandetanib was interrupted temporarily until the blood pressure decreased to grade ≤1 on amlodipine. Everolimus was continued at the same dose. Other toxicities were transient and manageable and did not require dose reductions or interruptions (Table 2).

Sequential MRI scans showed slow but steady local tumor progression (Fig. 5B). During cycle 7, the child developed increased slurred speech and ataxia, and vandetanib/everolimus was permanently discontinued 11 months after diagnosis. No further systemic treatment or reirradiation was administered. She then unexpectedly entered a “honeymoon period” of 8 months, during which the steroids could be stopped and her balance and activity levels went back to baseline. But eventually, she developed again worsening slurred speech and ataxia. MRI confirmed tumor progression locally and a metastatic deposit in the left lateral ventricle. The child died a month later, with an OS of 20 months from initial diagnosis.

The family generously donated the patient's brain tissue for postmortem study (Fig. 5C), whereby tumor cells were found to have infiltrated throughout the brainstem (Fig. 5D). In addition to the *HIST2H3C* and *ACVR1* mutations, which were found in all parts of the specimen, we observed the acquisition of a *KRAS*^G12A^ mutation, not found in the diagnostic biopsy specimen, at high frequency in the main body of the tumor in the inferior left pons and, to a lesser extent, in the right pons and superior specimens (Fig. 5E). In contrast to a specimen taken from the cerebellum without tumor cell infiltration, these pontine regions were found to have substantially elevated MAPK signaling by quantitative capillary electrophoresis for phospho-ERK1/2^T202/Y204^ (Fig. 5F). This mutation is known to confer resistance to EGFR and other RTK inhibitors, such as vandetanib (35, 36), and provides a rational explanation for eventual treatment failure in this case.

#### Case 2

A 4-year-old female was diagnosed with DIPG in July 2019 at Hospital Sant Joan de Déu (Barcelona, Spain). A biopsy was performed as part of an institutional precision medicine program. She received standard focal photon beam radiotherapy. Soon after radiotherapy, she developed hydrocephalus and required a ventriculo-peritoneal shunt followed by bevacizumab due to suspected pseudoprogression. After 2 months of bevacizumab and having identified an *ACVR1_*G328E mutation, treatment was switched to vandetanib 65 mg/m^2^/d and sirolimus 3 mg/m^2^/d. After one cycle of vandetanib/sirolimus, she developed increased ataxia, abnormal ocular movements, and akathisia. A head CT scan showed an intratumoral hemorrhage, and vandetanib/sirolimus was permanently discontinued. Neurologic symptoms persisted despite steroids, and she developed severe neuropsychological agitation and polyphagia as a result of prolonged use of steroids. Hence, 1 month after stopping vandetanib/sirolimus and following stabilization of the intratumoral hemorrhage, bevacizumab was restarted as steroid-sparing agent. The child could be weaned off steroids, and her restlessness resolved. After 2 additional months on bevacizumab, she experienced further clinical progression with stable MRI findings. She died a month later, with an OS of 9 months from initial diagnosis.

#### Case 3

A 4-year-old female was diagnosed with DIPG in January 2019 at Gustave Roussy (Paris, France). A biopsy was performed as part of the BIOMEDE trial (NCT002233049). She received standard focal photon beam radiotherapy together with dasatinib, showing clinical and radiologic improvement at the end of radiotherapy. Eventually, she developed worsening symptoms as a result of necrotic degeneration of the tumor, and bevacizumab was started due to suspected pseudoprogression. After 2 months on bevacizumab, steroids were stopped. In September 2019 (8 months after biopsy), she experienced tumor progression and hydrocephalus, requiring a ventriculo-peritoneal shunt. Based on a *PIK3CA* mutation, dasatinib was switched to sirolimus, and she underwent reirradiation (18 Gy in 2 weeks) in November 2019. Following a transient improvement, she developed further clinical deterioration and received two doses of bevacizumab, followed by tapering of the steroids, while continuing with sirolimus. With her tumor harboring an *ACVR1*^G328E^ mutation, in April 2020, treatment was electively switched to combined vandetanib/everolimus (dosing as per case 1). After 1 week on treatment, she developed hypertension grade 1 (no antihypertensives were started). She then progressively developed truncal hypotonia, and 17 days after the start of vandetanib/everolimus, she was admitted with headaches and vomiting. A noncontrast head CT scan showed increased size of the brainstem with associated intratumoral hemorrhage. Despite mannitol and high-dose steroids, she deteriorated rapidly and died within 24 hours of admission.

#### Case 4

A 4-year-old male diagnosed with DIPG in January 2019 was treated with cisplatin/temozolomide followed by standard focal radiotherapy and followed shortly after by ONC201. He underwent a biopsy in April 2019 that identified *HIST1H3B*^K27M^ and *ACVR1*^G328E^ mutations. Between July and August 2019, he received three doses of nivolumab. In September 2019, vandetanib/everolimus was started in Zurich, Switzerland. Treatment was discontinued within 10 weeks due to issues with compliance. His MRI post-vandetanib/everolimus showed significant inflammatory changes and cystic degeneration, which were challenging to interpret. The family subsequently pursued treatment overseas, and the child was lost to follow-up. He died 13 months after initial diagnosis.

## DISCUSSION

With ACVR1 representing a promising therapeutic target for a significant proportion of patients with DIPG, the need for translation of appropriate therapeutics is a high priority. In addition to specific approved inhibitors currently in development, repurposing of agents represents an attractive and more expedited option. A key criterion for drug repurposing is to identify agents active against the receptor at concentrations likely to be achieved within the tumor cells of patients with DIPG. Here we have used a proprietary AI-based platform to identify a novel combination of the multi-RTK inhibitor vandetanib alongside the mTOR inhibitor everolimus, taking advantage of the abilities of the latter drug to inhibit both mTOR and the ABC efflux transporter pumps, which has previously limited clinical utility of the former compound against CNS tumors.

We validated the ability of vandetanib to directly inhibit both wild-type and mutant ACVR1 signaling and in particular noted it be to more potent in this context than other purported kinase inhibitors such as dasatinib (29) and crizotinib (30). Although highly pleiotropic, we provide clear biochemical data that vandetanib inhibits both mutant and wild-type ACVR1, as well as a modest (approximately twofold) although significantly increased potency in heterogeneous *ACVR1*-mutant patient-derived DIPG cells compared with wild-type. In these models, the combination was synergistic *in vitro* and extended survival *in vivo*. Although vandetanib does not have the potency of other more selective agents against ACVR1 (12), its ability to inhibit the VEGFR pathway makes it additionally attractive in this context given the reported ability of mutant *ACVR1* to drive angiogenesis in DIPG (14). Similarly, the use of an mTOR inhibitor in combination is strongly suggested in this genotype, given the significant cosegregation of genetic alterations targeting the PI3K pathway in *ACVR1*-mutant tumors (*PIK3CA*, *PIK3R1*, and *PTEN*; refs. 12, 37). Importantly, we and others have shown combined targeting of ACVR1 and mTOR to be synergistic *in vitro* using both tool compounds and novel ACVR1 inhibitors in patient-derived DIPG models (ref. 38; https://m4kpharma.com/recording-of-m4k-pharmas-scientific-update-meeting-june-12th-2019/). Inhibition of the downstream SMAD target ID1 by vandetanib was also markedly more pronounced in the presence of everolimus, reflecting previous reports of the regulation of ID1 by mTOR (39) and presumably further underlying the synergistic effect we observed *in vitro.*

Vandetanib, an established inhibitor of VEGFR, EGFR, and RET kinases, is approved for the treatment of certain thyroid cancers. In common with many kinase inhibitors, vandetanib has been reported to have limited CNS penetration, as it is a substrate of ABC transporter pumps that limit crossing of small molecules across the blood–brain barrier (40). To overcome this, the use of everolimus to inhibit ABCB1 (P-gp) and ABCG2 (BCRP) has been shown to result in significantly increased vandetanib brain concentrations in mice (41). In adults, a patient with a *KIF5B–RET* fusion–positive non–small cell lung cancer (NSCLC) and brain metastases showed a decrease in the intracranial disease burden upon treatment with vandetanib plus everolimus (33) as part of the phase I clinical trial in patients with advanced or metastatic cancer (NCT01582191). Here, fatigue, rash/acne, diarrhea, mucositis, hyperglycemia, hypertriglyceridemia, and hypercholesterolemia were the most common toxicities, and 6 of 80 (7.5%) patients experienced dose-limiting toxicities (DLT; ref. 42). The MTDs and recommended phase II doses (RP2D) were defined as 300 mg once daily for vandetanib and 10 mg once daily for everolimus. Activity in terms of reduction of tumor volumes was noted to be significantly enhanced in patients with molecular alterations in the drug targets (RET, VEGFR, EGFR, and PI3K/mTOR), supporting further development of the combination (42).

Given the urgent clinical need, we report the first use of this combination in children with DIPG treated in four expert pediatric neuro-oncology centers across Europe to provide preliminary evidence of the dosing and toxicity profile of vandetanib/everolimus, which may serve as a reference for future clinical trials. Importantly, two of four patients treated with this combination developed an intratumoral hemorrhage (ITH) during the first cycle of vandetanib/mTOR inhibitor. ITH may occur in 14% to 19% cases of DIPG (43) and tends to be associated with underlying tumor progression. One of these patients had a previous episode of pseudoprogression prior to vandetanib/sirolimus, and the other had two reported episodes of pseudoprogression plus one confirmed tumor progression prior to vandetanib/everolimus. Pseudoprogression rates in children with DIPG vary between 14% and 24%, and diagnosis can only be made retrospectively (44, 45). Hence, these episodes of pseudoprogression could have indeed represented genuine tumor progression. Notwithstanding, increased risk of bleeding is a known side effect of antiangiogenic drugs (46). Of note, among 60 children with DIPG treated with vandetanib ± dasatinib (28, 32), no episodes of intratumoral hemorrhage were reported, but there were four episodes of gastrointestinal hemorrhage (three grade 3 and one grades 1–2)—all of them in patients who had received both vandetanib/dasatinib. In our case series, both cases with ITH had received bevacizumab prior to vandetanib. Hence, an additive class effect cannot be ruled out, as treatment with bevacizumab is itself a known risk factor for CNS hemorrhage (46, 47), and so exclusion of patients with previous antiangiogenic therapies may need to be considered; the increased CNS penetration of vandetanib when combined with everolimus could have also led to enhanced on-target antiangiogenic effects in this context.

The other side effects of vandetanib and everolimus observed in three clinically annotated cases were within the toxicity profile reported for this class of compounds (28, 32, 48). One child developed hypertension grade 3 attributable to vandetanib, which subsequently resolved with temporary discontinuation of the drug and initiation of antihypertensive medication. Survival outcomes for the reported cases were largely within those previously reported in historical cohorts of children with DIPG (43).

Vandetanib has been tested clinically in patients with DIPG previously in two phase I trials, both as single agent (32) and combined with dasatinib (28). A total of 35 patients received single-agent vandetanib during and after radiotherapy (32). The MTD was not reached, with only one DLT (diarrhea grade 3) at the highest dose level, and 145 mg/m^2^/d was declared as the RP2D. Nevertheless, in the expansion phase, two patients on high doses of dexamethasone concurrently experienced grade 4 hypertension and posterior reversible encephalopathy syndrome during the first 10 days of treatment (one at 145 mg/m^2^/d and the other at 110 mg/m^2^/d). Additionally, other toxicities in patients treated with 110 to 145 mg/m^2^/d included grade 3 hypertension, hypokalemia, and photosensitivity (*n* = 1 each) and grade 1–2 QTc prolongation (*n* = 6). There were no objective responses. The 2-year OS was 21.4% (32). Unfortunately, no tumor tissue was available at diagnosis for molecular analysis except for one case.

In the phase I trial of dasatinib/vandetanib, 25 patients received the combination during and after radiotherapy. DLTs included grade 3 elevated amylase (*n* = 2) and elevated lipase, hypoalbuminemia, thrombocytopenia, and diarrhea (*n* = 1 each; ref. 28). The MTD of combined vandetanib and dasatinib was 65 mg/m^2^/d for each drug. Outside of the DLT evaluation period, the following relevant toxicities were reported: grade 4 anemia (*n* = 2); neutropenia, thrombocytopenia, hypokalemia, and infection (*n* = 1 each); grade 3 hypoalbuminemia (*n* = 5), neutropenia, hypokalemia, diarrhea and infection (*n* = 4 each); gastrointestinal hemorrhage (*n* = 3); hypertension and hypophosphatemia (*n* = 2 each); and prolonged QTc, proteinuria, fatigue, vomiting, and anemia (*n* = 1 each). All patients experienced progression with a 2-year OS of 9%.

Notably, among other RTKs, vandetanib inhibits EGFR. In our most extensively studied case, for which good quality of life was sustained for long periods prior to the child dying of the disease, a *KRAS*^G12A^ mutation was discovered at autopsy. This mutation has been shown to confer resistance to EGFR (49–51) and other RTK inhibitors, including vandetanib (35, 36), in NSCLC. Although highlighting the likely acquisition of genetic alterations that allow treatment escape with any targeted inhibitor, these data are also strongly suggestive that vandetanib was reaching the tumor in sufficient quantities to exert a growth inhibitory effect. In previous clinical trials with vandetanib in patients with DIPG, pharmacokinetic studies found a cerebrospinal fluid–to–plasma ratio for vandetanib of approximately 2% for two patients treated at 65 mg/m^2^/d (28), suggesting a substantial improvement for our proposed combination.

Everolimus also represents an excellent backbone for combinatorial clinical studies in this disease given the wealth of safety data in patients with DIPG (NCT02233049, NCT03352427, and NCT0335579) and its demonstrated safety and tolerability in the BIOMEDE trial (NCT02233049). The experience of everolimus in this age group may be of particular importance given the reservations expressed about the ability to provide clinical doses of the drug at sufficient concentrations to elicit significant inhibition of P-gp (52). Notably, another recent study reported the use of everolimus to improve the CNS penetration of dasatinib in PDGFRA-driven high-grade gliomas in children (53). Here, the authors found an increase in both plasma (11.2–31.5 ng/mL) and CSF (0.44–0.91 mg/mL) dasatinib levels in two patients after 1 week of dasatinib monotherapy and then after two cycles of dual therapy with dasatinib and everolimus (53), as well as pointed to adult phase I studies of higher doses of everolimus (up to 70 mg weekly) that were well tolerated and resulted in mean peak levels of everolimus tenfold higher than standard regimens (54).

In summary, vandetanib and everolimus is a feasible combination to trial for children with *ACVR1*-mutant DIPG, further mandating the inclusion of stereotactic and/or liquid biopsy approaches for molecular profiling to guide biology-driven stratified trials (55). Following treatment-induced changes by molecular profiling of sequential liquid biopsies and/or postmortem specimens will help improve our understanding of clonal evolution and mechanisms of resistance to such approaches. Going forward, a formal early-phase clinical trial with standardized safety and efficacy monitoring will be essential to determine the clinical utility of such an approach for children with these tumors.

## METHODS

### Identification of Therapeutic Combinations

The Benevolent platform ingests and processes structured and unstructured data to create a proprietary knowledge graph comprising 1.2 billion relationships (gene–disease, gene–drug, drug–disease, and gene–gene connections), 95% of which are extracted from unstructured data (i.e., scientific literature) and harmonizes biomedical data to build representations of disease biology. The graph is constantly being updated and curated in the light of new discoveries through machine reading. Inferences are drawn and relationships inferred between the entities in the graph through the use of proprietary AI algorithms, including Rosalind (26), that combine relational inference via tensor factorization with graph-based data integration to predict disease genes and potential points of therapeutic intervention. The knowledge graph extracts data from heterogeneous sources, including literature evidence, differential expression, and clinical trial data, and consists of entities connected through relationships (e.g., therapeutic relationship or biological association). In Rosalind, a tensor factorization model is trained on this heterogeneous knowledge graph to produce a ranked list of genes as targets for disease interventions. Rosalind uses a state-of-the-art scoring function that enables the modeling of asymmetric relationships between entities. For DIPG, the graph was queried in the search for an approved drug capable of inhibiting one or more of the mutated putative drivers of this disease. In light of the poor brain penetration of the identified drugs, the graph was queried again in the search for a combination of drugs that had already been dosed in humans and that, through modulation of the drug efflux pumps, could increase the brain penetration.

### Generation of Wild-Type and Mutant ALK2 Constructs

All constructs (GST-6His-ALK2 variants) were optimized for expression in insect cells and were cloned into pOET1 using the flashBAC system (Oxford Expression Technologies). Sf9 cells were transfected with the *ALK2* gene in pOET1 and flashBAC baculoviral genome. All constructs were expressed at a 300-mL scale, and proteins were purified using microscale affinity columns (glutathione or Ni-NTA Phynexus tips). Enzyme activity was measured by the ADP-Glo Kinase Assay (Promega).

### Caco-2 Permeability

Apparent permeability (*P*_app_) was determined in the Caco-2 human colon carcinoma cell line. Cells were maintained (DMEM with 10% FBS, penicillin, and streptomycin) in a humidified atmosphere with 5%CO_2_/95% air for 10 days. Cells were plated out onto a cell culture assembly plate (Millipore), and monolayer confluency was checked using a transepithelial electrical resistance (TEER) electrode prior to the assay. Media were washed off and replaced in the appropriate apical and basal wells with Hank's Balanced Salt Solution buffer (pH 7.4). Bidirectional A–B and B–A apparent permeabilities of the test compounds vandetanib and everolimus were determined in the presence of E3S [estrone-3-sulfate, an ABCG2 (BCRP) substrate] or indinavir [an ABCB1 (P-gp) substrate]. LY335979 (P-gp inhibitor) and Ko134 (BCRP inhibitor) were used as positive controls. The Caco-2 plate was incubated for 2 hours at 37°C in a humidified atmosphere with 5% CO_2_, and Lucifer yellow was used to confirm membrane integrity after the assay. Samples from the apical and basolateral chambers were analyzed using reverse chromatography tandem mass spectrometry on a Waters TQ-S.

### Cell Culture

All patient-derived material for cell culture was collected under Research Ethics Committee approval from the originating centers. Cells were grown in stem cell media consisting of DMEM/Nutrient Mixture F12, Neurobasal-A Medium, HEPES Buffer Solution 1 mol/L, sodium pyruvate solution 100 nmol/L, nonessential amino acids solution 10 mmol/L, Glutamax-I Supplement, and Antibiotic–Antimycotic solution (all Thermo Fisher). The media were supplemented with B-27 Supplement Minus Vitamin A (Thermo Fisher), 20 ng/mL Human EGF, 20 ng/mL Human FGF–Basic 154, 20 ng/mL Human PDGF-AA, 20 ng/mL Human PDGF-BB (all Shenandoah Biotech), and 2 μg/mL Heparin Solution (0.2%, Stem Cell Technologies). Cell authenticity was verified using short tandem repeat DNA fingerprinting.

### Compound Efficacy Assays

Cells were plated at a density of 2,000 to 4,000 cells/well on laminin-coated 96-well plates in a minimum of triplicates. After 3 days of incubation, compounds were added to each well and incubated at 37°C in 5% CO_2_, 95% humidity for 8 days (192 hours). Drugs were combined by adding one compound in rows and another in columns with serial dilutions, resulting in a 6 × 9 dose matrix. Vandetanib and everolimus were purchased from Selleckchem. Cell viability was assessed by the CellTiter-Glo luminescent cell viability assay (Promega). SynergyFinder (https://synergyfinder.fimm.fi) was used for interactive analysis and visualization of drug combination profiling data following the Bliss independence model (56).

### Western Blot Analysis

For treatment with vandetanib and everolimus, cells were incubated in complete media with vehicle or increasing concentrations of drug (0.1, 1, 10 μmol/L), and protein was collected at 4 and 8 hours posttreatment. Mouse brain samples were manually homogenized in protein cell lysis buffer. Samples were lysed by using lysis buffer (Cell Signaling Technology) containing phosphatase inhibitor cocktail (Sigma) and protease inhibitor cocktail (Roche Diagnostics). Following quantification using the Pierce BCA Protein Assay Kit (Thermo Fisher), equal amounts of cell extracts were loaded for Western blot analysis. Membranes were incubated with primary antibody (1:1,000) overnight at 4°C and horseradish peroxidase secondary antibody (Amersham Bioscience) for 1 hour at room temperature. Signal was detected with ECL Prime Western blotting detection agent (Amersham Biosciences), visualized using Hyperfilm ECL (Amersham Biosciences), and analyzed using an X-ray film processor in accordance with standard protocols. Primary antibodies used were phospho-SMAD1/5/8 (CST#13820), phospho-AKT (Ser473, CST#4060), total AKT (CST#9272), and GAPDH (CST#2118), all Cell Signaling Technology, and SMAD1/5/8 (SC#6031) and ID1 (SC#488), both Santa Cruz Biotechnology.

### Tolerability and Pharmacokinetics

NOD.SCID animals were treated with an oral dose of everolimus (5 mg/kg) or vehicle (10% w/v hydroxypropylbetacyclodextrin in PBS), followed 30 minutes later by an i.p. dose of vandetanib (25 mg/kg) or vehicle during 14 consecutive days. Plasma and brain tissue samples were taken in triplicate at 1 hour after the last dose. Analysis was carried out by LC-MS/MS using a Xevo TQS coupled with an Acquity UPLC H-class system (Waters). Chromatography was carried out using a Phenomenex C18 X-B column (2.6 μm, 50 × 2.1 mm). Data acquisition was performed using Targetlynx, version 4.1, and modeling was carried out using Phoenix WinNonlin version 6.3.

### *In Vivo* Efficacy Studies

All experiments were performed in accordance with institutional and European guidelines (EU Directive 2010/63/EU) and were approved by the local animal care and use committee (Comite Etico de Experimentacion Animal at Universidad de Barcelona, protocol 135/11) or the UK Home Office Animals (Scientific Procedures) Act 198 and the United Kingdom National Cancer Research Institute guidelines for the welfare of animals in cancer research. Both patient-derived *H3F3A*^K27M^, ACVR1^R206H^ HSJD-DIPG-007 cells and those derived from a Nestin-Tv-a; Trp53^fl/fl^; Hist1h3b^K27M^, Acvr1^R206H^ (14) GEMM were used for orthotopic implantation. A single-cell suspension of each culture was made the day before implantation and cultured overnight. On the implantation day, small tumorspheres in exponential growth were harvested by mild centrifugation. Three-week-old female athymic mice were anesthetized with 100 mg/kg ketamine and 10 mg/kg xylazine and immobilized in a stereotaxic apparatus (Stoelting) at coordinates x+0.5 and y−5.4 from the bregma suture. HSJD-DIPG-007 tumorspheres (5 × 10^5^ cells), suspended in 5 μL Matrigel (BD Biosciences), were injected at a 3.1-mm depth (targeting the fourth ventricle) with a dull 22-gauge needle attached to a 50-μL syringe (Hamilton), using a stereotaxic arm. The animals (*n* = 72) were divided in four groups, with treatment starting 4 weeks (day 25) after tumor inoculation. The treatment arms comprised the following: group 1, DMSO-saline vehicle, *n* = 22; group 2, 25 mg/kg/d vandetanib (i.p.) daily for a 5-day period for 4 consecutive weeks, *n* = 16; group 3, 5 mg/kg/d everolimus (orally) daily for a 5-day period for 4 consecutive weeks, *n* = 17; and group 4, 25 mg/kg/d vandetanib (i.p.) and 5 mg/kg/d everolimus (orally), both daily for 5 days on, 2 days off per week for 4 consecutive weeks, *n* = 17. Everolimus was dosed 30 minutes before vandetanib. Mice were monitored by daily weighing and sacrificed by decapitation upon deterioration of condition or 20% weight loss from the maximum weight achieved, with tissue taken for further analysis. Mouse brains collected at the end of the efficacy study were processed for IHC. Plasma and brain samples from treated and control mice were taken at 2 hours postdose at the end of the 4-week treatment for pharmacokinetic and pharmacodynamic analyses.

### IHC

Paraformaldehyde-fixed mouse brains were paraffin embedded and sectioned (4 μm) for IHC analysis and stained with hematoxylin and eosin. For IHC, sodium citrate (pH 6.0) heat-mediated antigen retrieval was performed, and staining was carried out using an antibody directed against human nuclear antigen (Millipore, #4383, 1:100). Pressure antigen retrieval was performed and staining was carried out using antibodies directed against Ki67, (DAKO, #7240, 1:100) and CD31 (Abcam, #28364, 1:50). All primary antibodies were diluted into 1% Tris buffer solution with 0.05% Tween-20, except Ki67, which was diluted into Dako antibody diluent. Antibodies were incubated for 1 hour at room temperature. A Novocastra Novolink Polymer Detection Systems Kit (Leica Biosystems RE-7150) was used for the staining. Slides were then mounted using a Leica CV Ultra mounting medium, and slides were imaged using the high-throughput scanning microscope AxioScan Z1 and quantified using Definiens software.

### ddPCR

ddPCR was carried out on genomic DNA extracted from formalin-fixed, paraffin-embedded (FFPE) rolls from sagittal cuts of brains used for the tumor burden study, using the QIAamp DNA FFPE Tissue Kit (Qiagen). Custom TaqMan-based quantitative PCR genotyping assays (Bio-Rad; Applied Biosystems) were designed to specifically detect the *ACVR1*^R206H^ mutation present in HSJD-DIPG-007 (forward primer, GAATTACCGACACACTCCAACAGT; reverse primer, CTCTGGTCTTCCTTTTCTGGTACAA). The Bio-Rad QX200 ddPCR system was used. DNA was randomly encapsulated into approximately 15,000 oil nanoliter-sized droplets, using the Automated Droplet Generator (Bio-Rad, QX200 AutoDG), containing ddPCR Supermix for probes (no dUTP; Bio-Rad, 1863024), genotyping assay (VIC, wild-type, AGTGGCTCGCCAGATT; FAM, mutant, CAGTGGCTCACCAGATT), water, and the DNA of interest. The PCR reaction was performed in a thermocycler, and plates were then placed on the droplet reader in which the droplets were streamed individually through a detector and signals from mutant-positive (FAM), wild-type (VIC), double-positive (FAM and VIC/HEX), and negative droplets (empty) were counted to provide absolute quantification of DNA in digital form. The mutant allele concentration (C_MUT_) and wild-type allele concentration (C_WT_) were calculated with Quantasoft Analysis Pro (Bio-Rad), and the concentration of human DNA was calculated as previously described (57, 58).

### Liquid Biopsy Extraction and Sequencing

Up to 10 mL of peripheral blood was collected into Cell-Free DNA Collection Tubes (Streck). Samples were centrifuged twice for 10 minutes—first at 1,600 × *g* and at up to 16,000 × *g* to remove cellular contents and/or debris. Cell-free DNA isolation from plasma was performed using the QIAamp circulating nucleic acid kit (Qiagen, 55114) following quantification using the Qubit fluorometer (Thermo Fisher Scientific, dsDNA HS Assay kit, Q32854) and fragment analysis by 2200 and 4200 TapeStation (Agilent, Genomic DNA ScreenTape 5067–5366).

### Capillary-Based Protein Quantification

Capillary electrophoresis was conducted on protein lysates from the DIPG autopsy specimens using the automated Wes system (Protein Simple) with the 12- to 230-kDa Separation module (SM-W004) and the anti-rabbit detection module (DM-001) following the manufacturer's instructions and analyzed with Compass software. The following primary antibodies were used: ERK1/2 (1:100, Cell Signaling Technology, 9102), phospho-ERK^T202/Y204^ 1:100 (Cell Signaling Technology, 9101), and α-actin (1:200, Cell Signaling Technology, 6487). Goat anti-rabbit HRP conjugate (ProteinSimple, 042–206) was used as a secondary antibody.

### Clinical Treatment with Vandetanib and mTOR Inhibitor

Four children with biopsy-confirmed *ACVR1*-mutant DIPG were treated with vandetanib and an mTOR inhibitor in separate European institutions. All interventions, carried out with written informed consent from the patients' families, were conducted in accordance with the Declaration of Helsinki and under Ethical Review Board approval. The indication to use this combination and the clinical monitoring were at the discretion of the treating physician. Efficacy and safety data were collected retrospectively from each center. Toxicity grading was performed as per Common Terminology Criteria for Adverse Events version 4.03 for consistency with the BIOMEDE trial (NCT02233049).

### Statistical Analysis

Statistical analysis was carried out using R (www.r-project.org) and GraphPad Prism (GraphPad Software). Comparisons between groups of continuous variables employed the Student *t* test or ANOVA. Univariate differences in survival were analyzed by the Kaplan–Meier method, and significance was determined by the log-rank test or median test. All tests were two-sided, and a *P* value of less than 0.05 was considered significant for simple comparisons. Bonferroni correction for multiple comparisons was applied for combinatorial experiments, with a *P* value of less than 0.0083 considered significant.

## Authors' Disclosures

P.J. Richardson is an employee of BenevolentAI. R. Stankunaite reports other support from the National Institute for Health Research (NIHR) Biomedical Research Centre at The Royal Marsden NHS Foundation Trust and Christopher's Smile during the conduct of the study. R. Ruddle reports grants from The Institute of Cancer Research during the conduct of the study. A. Donovan reports grants from The Institute of Cancer Research during the conduct of the study. A. Pal reports grants from The Institute of Cancer Research during the conduct of the study. J. Grill reports nonfinancial support from Novartis outside the submitted work. M. Hubank reports personal fees from Novartis and AstraZeneca outside the submitted work. F. Carceller reports grants from the Giant Pledge via The Royal Marsden Cancer Charity and other support from GlaxoSmithKline outside the submitted work. C. Jones reports grants from Brain Research UK, the DIPG Collaborative, Children with Cancer UK, Abbie's Army, Lucas's Legacy, the Lyla Nsouli Foundation, and Cancer Research UK during the conduct of the study. No disclosures were reported by the other authors.

## Supplementary Material

Supplementary Figures S1-S3 Supplementary Figures S1-S3
