# Supplementary material for: Repurposing Vandetanib plus Everolimus for the Treatment of ACVR1-Mutant Diffuse Intrinsic Pontine Glioma
Source: Cancer Discov. 2021 Sep 22;12(2):416–31. doi: 10.1158/2159-8290.CD-20-1201 (PMC7612365; doi:10.1158/2159-8290.CD-20-1201)
Supplement: Supplementary Figures S1-S3 [file cd-20-1201_supplementary_figures_s1-s3_suppsf1.pdf]

Supplementary figures for

Combined vandetanib and everolimus, identified by an artificial intelligence platform, for the treatment of *ACVR1*-mutant diffuse intrinsic pontine glioma

Diana M Carvalho, Peter J Richardson, Nagore Olaciregui, Reda Stankunaite, Cinzia Lavarino, Valeria Molinari, Elizabeth A Corley, Daniel Smith, Ruth Ruddle, Adam Donovan, Akos Pal, Florence I Raynaud, Sara Temelso, Alan Mackay, John P Overington, Anne Phelan, David Sheppard, Andrew Mackinnon, Bassel Zebian, Safa Al-Sarraj, Ashirwad Merve, Jeremy Pryce, Jacques Grill, Michael Hubank, Ofelia Cruz, Andres Morales La Madrid, Sabine Mueller, Angel M Carcaboso, Fernando Carceller and Chris Jones

Supplementary Figure S1

A

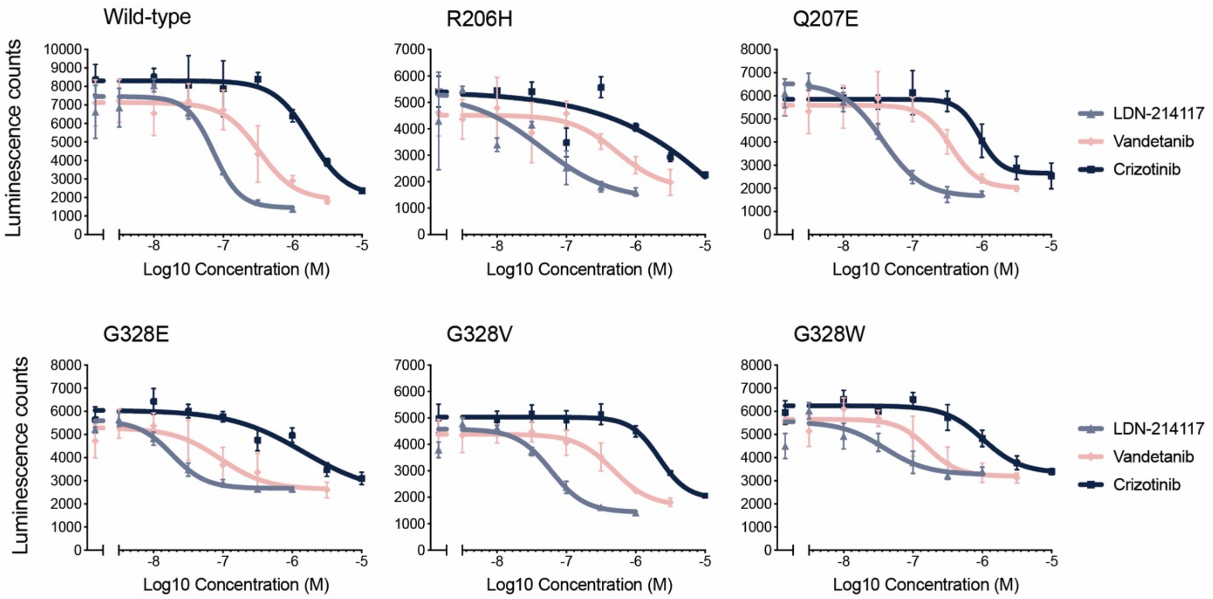

Supplementary Figure S1 – *Effects on wild-type and mutant ACVR1 of various targeted inhibitors.* Dose-response curves for LDN-214117 (grey), vandetanib (pink) and crizotinib (dark blue) against kinase activity of ACVR1 wild-type, R206H, Q207E, G328E, G328V and G328W mutations. Concentration of compound is plotted on a log scale (x axis) against luminescence counts (y axis).

Supplementary Figure S2

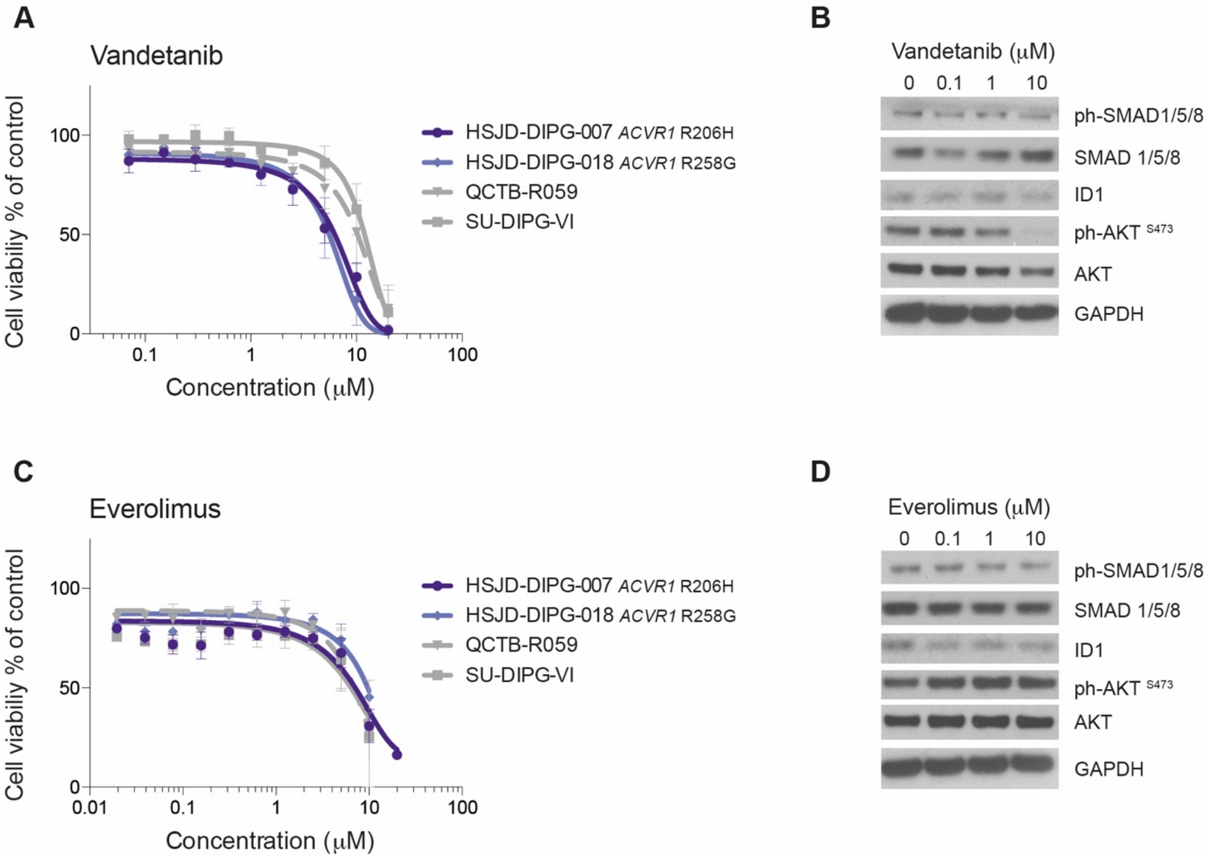

Supplementary Figure S2 – *Effect of single agent vandetanib and everolimus on DIPG cells in vitro.* (A) Concentration-response curves for vandetanib tested against two *ACVR1* mutant cell cultures (HSJD-DIPG-007 (R206H), HSJD-DIPG-018 (R258G), purple) and two wild-type cultures (SU-DIPG-VI, QCTB-R059, grey). (B) Western blot of *ACVR1*_R206H HSJD-DIPG-007 cells treated with increasing concentrations of vandetanib. GAPDH is the loading control. (C) Concentration-response curves for everolimus tested against two *ACVR1* mutant cell cultures (HSJD-DIPG-007 (R206H), HSJD-DIPG-018 (R258G), purple) and two wild-type cultures (SU-DIPG-VI, QCTB-R059, grey). (D) Western blot of *ACVR1*_R206H HSJD-DIPG-007 cells treated with increasing concentrations of everolimus. GAPDH is the loading control.

Supplementary Figure S3

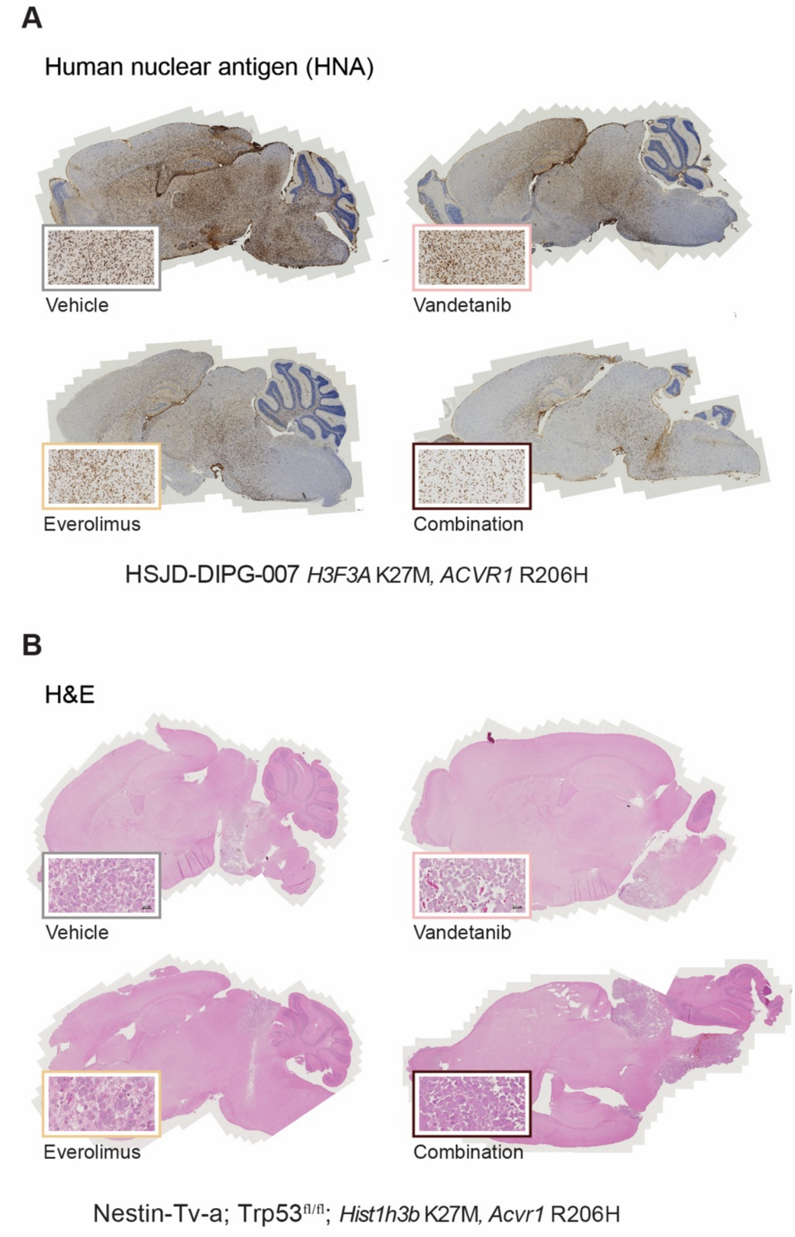

Supplementary Figure S3 – *Assessment of PDX and GEMM models treated with vandetanib and everolimus in vivo.* (A) Immunohistochemistry for anti-human nuclear antigen (HNA) of HSJD-DIPG-007 mice treated with vehicle control (grey), vandetanib (pink), everolimus (light orange), or the combination (dark red). Scale bar = 1000μM. (B) Haematoxylin and eosin (H&E) staining of Nestin-Tv-a; Trp53$^{fl/fl}$; Hist1h3b_K27M, Acvr1_R206H mice treated with vehicle control (grey), vandetanib (pink), everolimus (light orange), or the combination (dark red). Scale bar = 1000μM.
